# Muscle Up: Male Athletes’ and Non-Athletes’ Psychobiological Responses to, and Recovery From, Body Image Social-Evaluative Threats

**DOI:** 10.1177/15579883231155089

**Published:** 2023-02-21

**Authors:** David M. Brown, Cameron Muir, Kimberley L. Gammage

**Affiliations:** 1 Faculty of Applied Health Sciences, Brock University, St. Catharines, Ontario, Canada; 2 Department of Psychology, Brock University, St. Catharines, Ontario, Canada; 3 Department of Kinesiology, Brock University, St. Catharines, Ontario, Canada

**Keywords:** athletes, body image, cortisol, men, shame, social self-perseveration theory, social-evaluative threats

## Abstract

Negative body image often occurs as a result of social evaluation of the physique in men. Social self-preservation theory (SSPT) holds that social-evaluative threats (SETs) elicit consistent psychobiological responses (i.e., salivary cortisol and shame) to protect one’s social-esteem, status, and standing. Actual body image SETs have resulted in psychobiological changes consistent with SSPT in men; however, responses in athletes have yet to be examined. These responses may differ as athletes tend to experience fewer body image concerns compared with non-athletes. The purpose of the current study was to examine psychobiological (i.e., body shame and salivary cortisol) responses to an acute laboratory body image SET in 49 male varsity athletes from non-aesthetic sports and 63 male non-athletes from a university community. Participants (age range 18–28 years) were randomized into a high or low body image SET condition, stratified by athlete status; measures of body shame and salivary cortisol were taken across the session (i.e., pre, post, 30-min post, 50-min post-intervention). There were no significant time-by-condition interactions, such that athletes and non-athletes had significant increases in salivary cortisol (*F*_3,321_ = 3.34, *p* = .02), when controlling for baseline values, and state body shame (*F*_2.43,262.57_ = 4.58, *p* = .007) following the high-threat condition only. Consistent with SSPT, body image SETs led to increased state body shame and salivary cortisol, although there were no differences in these responses between non-athletes and athletes.

Body image is defined as a multidimensional construct involving perceptions and attitudes (i.e., cognitions, emotions, behaviors) regarding the body’s physical appearance and functional capabilities (Cash, 2012). Although researchers have primarily examined negative body image in young adult women, in the past decade, research has provided evidence to support the idea that young adult men are concerned about their bodies and experience body dissatisfaction and shame to a significant degree (Fiske et al., 2014; Griffiths et al., 2016). It has been suggested that men underreport their body image concerns due to societal stigma surrounding body image as a primarily women’s concern (Lamarche et al., 2018). It is likely that men experience greater levels of negative body image than previously reported, which may result in them engaging in behaviors (e.g., diet, exercise) as a way of obtaining and maintaining the muscular ideal frequently perpetuated by family, peers, and through popular media within Western society (Tiggemann, 2011). Given the impact of negative body image on self-esteem (van den Berg et al., 2010), anabolic steroid use (Walker et al., 2009), and psychological distress (Griffiths et al., 2016), it is crucial that researchers continue to investigate this construct and its consequences in men.

Although athletes have generally reported less negative body image compared with exercisers or non-athletes (Hausenblas & Symons Downs, 2001), Sabiston et al. (2019) suggested that the relationship between sport and body image is complex, potentially due to the sport environment, which highlights both the body’s appearance and functional capabilities. In addition to sociocultural pressures from family, friends, and popular media to uphold an unrealistic muscular ideal, athletes also experience sport-specific body pressures from coaches, teammates, and trainers to obtain and maintain a body that is conducive to their respective sport (Petrie & Greenleaf, 2012; Tiggemann, 2011). Often, these two ideals (muscular vs. sport-specific) conflict and can result in greater levels of body dissatisfaction for athletes (Petrie & Greenleaf, 2012). Indeed, research supports the idea that the relationship between body image and sport is more complex than previously thought. For example, researchers have reported that despite having an appreciation for the body’s functional capabilities, male athletes still express concerns over their physical appearance and note pressures from coaches, teammates, and peers to meet and uphold society’s muscular ideal (Galli & Reel, 2009; Lunde & Gattario, 2017; Tiggemann, 2011). These body image concerns may in part be due to the type of sport, as researchers have identified that athletes in weight-dependent and appearance-focused sports report higher levels of disordered eating and negative body image, and lower levels of positive body image compared with athletes in non-appearance focused sports (Chapman & Woodman, 2016; Varnes et al., 2013). Due to the many harmful health-related consequences that can stem from negative body image, and the complex relationship that exists between body image and sport, it is important to continue to investigate the relationship between these two constructs.

One theory that has been useful in understanding negative body image is social self-preservation theory (SSPT; Dickerson et al., 2004; Dickerson & Kemeny, 2004; Kemeny et al., 2004). SSPT holds that humans have an innate need to belong to and be accepted by others. It is important for people’s social identity (e.g., social esteem, status, and standing) to be held in high regard and viewed favorably by others in their social groups (Kemeny et al., 2004). Imaginary, potential, and actual social-evaluative threats (SETs) may pose a risk to one’s social identity by calling into question competencies regarding certain characteristics, particularly those people regard as being important to their social identity (Dickerson et al., 2004; Dickerson & Kemeny, 2004; Kemeny et al., 2004). Researchers have reported that psychological (e.g., self-conscious emotional responses, particularly shame) and physiological (e.g., cortisol) reactions occur in response to psychosocial stressors that challenge the social-self (Dickerson et al., 2004; Dickerson & Kemeny, 2004; Kirschbaum et al., 1993). In a meta-analysis of 208 studies, Dickerson and Kemeny (2004) examined the effect of acute laboratory stressors on cortisol responses. They reported that stressors involving social evaluation, where the outcome of the task was uncontrollable (i.e., participants would perform poorly on the task no matter what), that involved a permanent record (e.g., videotaping), and that involved characteristics of importance to the individual yielded the greatest cortisol response (Dickerson & Kemeny, 2004). It was proposed that these psychobiological responses serve to warn the individual of the potential threat so that they can engage in various behaviors (e.g., appeasement, withdrawal, avoidance) to reduce the threat and avoid damage to the social-self (Dickerson et al., 2004; Dickerson & Kemeny, 2004; Kemeny et al., 2004).

Researchers have frequently utilized the Trier Social Stress Test (TSST; Kirschbaum et al., 1993) to induce psychosocial stress. In this paradigm, participants are given 5 min to prepare and deliver a 5-min speech in front of a panel of judges, which is followed by performing a series of mathematical problems. Consistently, the TSST has led to increases in shame and cortisol, consistent with SSPT (Dickerson & Kemeny, 2004). Only a few studies have investigated psychobiological responses to SETs in athletes. Rimmele et al. (2007) investigated changes in heart rate, salivary cortisol, anxiety, and mood (i.e., calmness) between national level endurance runners and non-exercisers (i.e., those who engage in two or fewer hours of exercise per week) in response to the TSST. They reported that the highly trained athletes exhibited significantly lower heart rate and salivary cortisol compared with the non-exercisers. In addition, mood and anxiety significantly increased in the non-exercisers following the TSST, but no changes were reported in the athletes. In a follow-up study, Rimmele et al. (2009) investigated the potential impact of competition level on athletes’ psychobiological responses (i.e., heart rate, salivary cortisol, state anxiety, and mood). In this study, Rimmele et al. (2009) divided their athletes into two groups (elite vs. amateur) based on objective measures of cardiorespiratory fitness (e.g., submaximal running velocity) and training hours per week. Rimmele et al. (2009) reported that elite sportsmen had significantly lower salivary cortisol, heart rate, and state anxiety compared with the non-active individuals and amateur sportsmen in response to the TSST. Although the amateur sportsmen had significantly lower heart rate compared with the non-active control group, there were no significant differences in salivary cortisol or state anxiety levels between athlete groups. Furthermore, while mood and calmness worsened as a result of the stressor for all groups, there were no significant between group differences observed. Rimmele et al. (2009) concluded that elite sportsmen reported reduced psychobiological responses to psychosocial stress, in the form of lower anxiety, heart rate, and salivary cortisol, compared with non-active individuals and amateur athletes.

Although these studies suggest that elite athletes exhibit blunted psychobiological responses to psychosocial stress compared with amateur sportsmen and non-exercisers, they neglected to measure changes in shame, which is the key emotional response according to SSPT. In addition, elite and amateur sportsmen were athletes from endurance-based sports only and were separated primarily based on cardiorespiratory fitness rather than competition level per se. Therefore, these findings may not reflect all athletes or athletes who compete at different levels of competition. Furthermore, while sport participation buffered psychobiological responses to a specific SET (i.e., TSST) in highly trained individuals compared with amateur sportsmen and untrained individuals (Rimmele et al., 2007, 2009), athletes may respond differently to other SETs that challenge more important aspects of their social self, such as social evaluation of the physique, especially given the additional social pressures athletes face to obtain “ideal” bodies (Petrie & Greenleaf, 2012). Currently, only two studies have investigated body image SETs in men, with neither investigating these responses in athletes.

Lamarche et al. (2017) examined body shame and cortisol responses to body image SETs in 66 university men. Participants were randomly assigned to a high- or low-SET condition. Participants in the high-threat condition underwent several anthropometric measurements (i.e., height, weight, skin folds, various circumference measurements) and a measure of overall strength taken by an attractive female confederate, while shirtless in front of two full-length mirrored walls. A male confederate, who met the North American appearance ideal for men posed as a second participant and had his measurements taken and read out loud prior to the actual participants’ measurements. The men were also told that they would be videorecorded while their measurements were being taken to review later for accuracy. Participants in the low-threat condition waited for 10 min before having the same anthropometric and strength measurements taken, in the absence of confederates and the video camera, away from mirrors, and while wearing a t-shirt. Men in the high-threat group reported higher levels of body shame and had higher salivary cortisol compared with men in the low-threat condition, consistent with SSPT. Although Lamarche et al. (2017) were the first to examine psychobiological responses to body image SETs in university men, they failed to examine the potential recovery, which may be an indication of the stress response system’s adaptiveness.

In response, Smyth et al. (2020) investigated body shame and cortisol responses to, and recovery from, a body image SET in men. They also examined the behavioral responses associated with social evaluation of the physique. Using the same experimental procedures as Lamarche et al. (2017), Smyth et al. (2020) reported significantly higher body shame and cortisol responses in the high-threat condition compared with the low-threat condition immediately following the body image SET and 30-min post-stressors onset. In addition, men in the high-threat group exhibited greater amounts of body shame and submissive behaviors (e.g., slumped posture, downward tilted head) compared with men in the low-threat condition. Finally, there were no significant differences between groups for body shame or cortisol 50-min post-stressor onset (i.e., recovery), indicating an adaptive recovery from the body image SET.

Although men have reported psychobiological responses to body image SETs, athlete status has yet to be examined within this context. Athletes have been excluded from studies involving body-specific SETs due to the unique relationship they have with their bodies (Petrie & Greenleaf, 2012). Researchers using non-body image SETs have provided evidence to support the idea that athlete status may moderate psychobiological responses (Rimmele et al., 2007, 2009). Indeed, based on studies by Rimmele et al. (2007, 2009), it is possible that athletes would have a blunted psychobiological response to body-specific SETs. On the contrary, additional social pressures for male athletes to uphold the muscular ideal due to their athletic involvement could lead to greater responses than non-athletes to body-specific SETs as appearance and functionality of the body may be a particularly salient concern to them and their identity (Petrie & Greenleaf, 2012). However, athletes have yet to be examined in this context.

Therefore, the purpose of the current study was to examine psychobiological responses to (i.e., cortisol and body shame), and recovery from, a social-evaluative body image threat in university men and examine if athlete status moderated these responses. Due to the blunted psychobiological responses seen in previous studies that investigated non-appearance focused sport athletes (i.e., endurance runners) compared with non-exercisers (Rimmele et al., 2007, 2009), it was hypothesized that there would be a three-way interaction between athletic status (athletes vs. non-athletes), condition (high- vs. low-threat), and time (pre-threat vs. post-threat vs. intermediate-threat vs. recovery) for cortisol and state body shame, such that there would be significant increases in state body shame (pre to post-threat), and salivary cortisol (pre to intermediate-threat) in the high-threat condition only, and that these increases would be greater in the non-athletes compared with the athletes. Furthermore, it was hypothesized that there would be no significant changes in state body shame or salivary cortisol for either athletes or non-athletes in the low-threat condition.

## Method

### Participants

In this study, non-athletes were defined as individuals who did not currently engage in any form of sport at any level of competition (e.g., recreational or intramural sports). Varsity athletes were defined as members of a university competitive club or varsity team (competing at the provincial or national level). Athletes were not eligible to participate if they competed in aesthetic (i.e., the outcome of the competition is directly related to the competitors’ physical appearance or body positioning/posture, such as cheerleading, figure skating, and gymnastics) or weight-dependent sports (i.e., those in which athletes compete within weight classes or categories, such as powerlifting, rowing, boxing, and wrestling) due to body image differences that may occur in athletes from these types of sports (Chapman & Woodman, 2016; Galli & Reel, 2009; Varnes et al., 2013).

Participants were not eligible to participate in the current study if they smoked, had a history/diagnosis of a clinical eating disorder, or if they had been diagnosed with a disease affecting cortisol, such as Cushing’s or Addison’s disease (Gold & Chrousos, 1985). Individuals were excluded from the study if they were taking any medication affecting cortisol, such as anti-depressants (Burke et al., 2005), or if they were using anabolic steroids. Eligible participants were emailed instructions 24 hr before their session reminding them to refrain from engaging in physical activity and consuming any food or beverages within 1 hr of the testing session due to their impact on salivary cortisol.

An a priori power analysis was performed to determine the required sample size needed for the proposed analyses. Based on medium to large effect sizes reported in previous studies utilizing similar procedures (Lamarche et al., 2017; Smyth et al., 2020; body shame η_p_^2^ = .10; body dissatisfaction η_p_^2^ = .17; cortisol η_p_^2^ = .11), the power analysis indicated that approximately 25 athletes and non-athletes were required per condition.

A total of 112 men between the ages of 18 and 28 who were either non-athletes (*n* = 63) or varsity/club athletes (*n* = 49) participated in the current study. Athletes reported competing in volleyball (*n* = 12; 24.49%), rugby (*n* = 9; 18.37%), and lacrosse (*n* = 5; 10.2%) most frequently. The remaining athletes came from a variety of other sports, including basketball (*n* = 3; 6.12%), cross-country (*n* = 3; 6.12%), track and field (*n* = 3; 6.12%), dragonboat (*n* = 3; 6.12%), fencing (*n* = 2; 4.08%), soccer (*n* = 2; 4.08%), ball hockey (*n* = 2; 4.08 %), baseball (*n* = 2; 4.08 %), tennis (*n* = 1; 2.04%), ultimate frisbee (*n* = 1; 2.04%), hockey (*n* = 1; 2.04%), and curling (*n* = 1; 2.04%). The most frequently self-reported ethnicity of the non-athletes was White (*n* = 38; 60.32%) followed by South Asian (*n* = 6; 9.52%), and Latino/Hispanic (*n* = 5; 7.94%). For the athletes, the most frequently self-reported ethnicity was also White (*n* = 38; 77.55%), followed by Black (*n* = 5; 10.2%). The remaining participants were relatively evenly divided between other ethnicities (e.g., Asian, Native American, Middle Eastern, mixed).

### Measures

#### Demographic Questionnaire

The demographic questionnaire screened participants for factors that could influence cortisol (e.g., antidepressant medication, hepatitis B) and was used to collect self-reported information, such as age, major, year of study, ethnicity, sexual orientation, and varsity/club sport (if applicable). Participants were screened for stressful events on the day of testing and to ensure that they followed the study requirements before arriving at the lab (i.e., refraining from eating, drinking, and exercising within 1 hr prior to the start of the session), as well as waking time that day.

#### Trait Questionnaires

The International Physical Activity Questionnaire-Short (Craig et al., 2003) was used to assess vigorous, moderate, and walking activity over the past 7-day period. Only total moderate and vigorous physical activity was analyzed in this study as they have been significantly related to positive physical and mental health outcomes in adults (World Health Organization [WHO], 2022). This is consistent with Canadian physical activity guidelines (Ross et al., 2020). Participants indicated the number of days per week, over the last 7-day period that they engaged in each intensity of physical activity for at least 10 min (WHO, 2022). Next, they recorded the typical duration for one of those bouts of physical activity. Total moderate-vigorous activity was calculated using the following formula: (Days Vigorous × Minutes Vigorous × 9) + (Days Moderate × Minutes Moderate × 5), based on metabolic equivalents (METs) for vigorous and moderate intensity activity.

The Sociocultural Attitudes Towards Appearance Questionnaire-4 (SATAQ-4; Schaefer et al., 2015) is a 22-item self-report questionnaire made up of five subscales. For the purpose of this study, only the subscale related to the internalization of the muscular/athletic ideal was used. Participants rated the 5 items on how much they agreed or disagreed with each of the statements ranging from 1 = *definitely disagree* to 5 = *definitely agree*. Mean scores were calculated and ranged from 1 to 5 with higher scores indicating higher levels of internalization of the muscular/athletic ideal. The muscular/athletic ideal subscale has shown adequate internal consistency ratings in U.S. males (α = .90; Schaefer et al., 2015). In the current study, the subscale showed good internal consistency ratings for the low (α_Muscularity_ = .82) and high-threat conditions (α_Muscularity_ = .80), respectively.

The Male Body Attitude Scale (MBAS; Tylka et al., 2005) is a 24-item self-report questionnaire with three subscales that measure attitudes about muscularity, low body fat, and height. Due to the importance placed on muscularity in men (Tiggemann, 2011), the current study used only the 10-item muscularity subscale. Participants rated the extent to which each statement applied to them on a 6-point scale, ranging from 1 = *never* to 6 = *always*. Means scores were calculated, with higher means scores indicating a greater desire for muscularity. The total MBAS, as well as the muscularity subscale has shown adequate internal consistency ratings (αMuscularity = .89 and αTotal = .91; Tylka et al., 2005). In the current study, the muscularity subscale of the MBAS showed good internal consistency ratings for the low (αMuscularity = .89) and high-threat conditions (αMuscularity = .89), respectively.

#### State Body Shame Questionnaire

The shame subscale of the Weight and Body-Related Shame and Guilt Scale (WBRSG-BS; Conradt et al., 2007) consists of six items that are rated from 0 to 4, with 0 = *strongly disagree* to 4 = *strongly agree* based on how the individual is feeling in that moment. Mean scores were calculated with higher scores indicating higher levels of state body shame. The original WBRSG-BS is a trait measure of body shame; however, it has previously been adapted as a measure of state body shame and has shown adequate internal consistency in college women (α = .81 and .92; Cloudt et al., 2014) and men (α = .91 and .94; Lamarche et al., 2017). In the current study, the internal consistency ratings for the state version of the WBRSG-BS ranged from acceptable to excellent for the low (α_time 1_ = .81; α_time 2_ = .81; α_time 3_ = .82; α_time 4_ = .85) and high-threat conditions at each time point (α_time 1_ = .78; α_time 2_ = .89; α_time 3_ = .90; α_time 4_ = .89), respectively.

### Manipulation Checks

#### Previous Anthropometric Experience

Participants were asked if they had any of the anthropometric or strength measurements taken previously. This measure was used to see if previously having undergone these measures could potentially explain any blunted responses observed.

#### Perceptions of Confederates

Participants in the high-threat condition were asked to rate how attractive they thought the female research confederate was and how similar the male confederate was to their muscular ideal. Participants rated the female confederate ranging from 0 = *not at all attractive* to 4 = *very attractive*, and the male confederate ranging from 0 = *not at all my perceptions of the muscular ideal* to 4 = *my exact perceptions of the muscular ideal* (Lamarche et al., 2017; Smyth et al., 2020).

### Procedure

Upon receiving ethics clearance from Brock University’s research ethics board (REB# 17-083), participants were recruited via classroom and sport team meeting announcements, and through a participant recruitment site run by the university’s psychology department. Prospective participants were told the study investigated mental health, hormones, and physical characteristics in university male athletes and non-athletes to obtain authentic responses, obscured from the anticipation of the SET (Lamarche et al., 2014; Martin Ginis et al., 2012). All participants provided written consent prior to their participation in the study.

All testing took place between 2 and 7 pm, when circulating cortisol is at its lowest and most stable for the day (Dickerson & Kemeny, 2004; Dunn et al., 1972). The testing location was a private room at the university with two mirrored walls. Testing took place across two sessions (separated by at least 48 hr) and participants were compensated US$10 CAD for their time at the end of each session. In the first session, participants completed the trait body image questionnaires as well as other mental health questionnaires used to disguise the true purpose of the study. Following the completion of the questionnaires, participants were provided with a written reminder of their next session time and the study requirements (i.e., refrain from eating, drinking, exercising within 1 hr of the session).

At the second session, participants underwent either the high or low SET condition. Upon arrival, participants were asked to change into their shorts using the private washrooms around the corner from the lab. Upon returning, participants were seated and provided a baseline saliva sample. Next, participants completed a baseline measure of state body shame. This took approximately 5 min to complete. Once completed, participants provided a second saliva sample (pre-threat). Following, participants were briefed on the series of physical measurements (chest, waist, and flexed biceps circumference, height, weight, percent body fat via skinfold measurements) and test of strength (handgrip) they would be undergoing. They then underwent their condition consistent with their group assignment (see below for descriptions). Immediately following the anthropometric and strength measures, a third saliva sample (post-threat), followed by the third state body shame questionnaire, was completed. Following a 10-min wait period, where the participants were instructed not to talk, sleep, or use any electronics, participants provided a fourth saliva sample (intermediate timepoint) followed by the state body shame questionnaire. This saliva sample was approximately 30-min post-stressor onset, which has been identified as the optimal time to capture peak cortisol responses to psychosocial stressors (Dickerson & Kemeny, 2004; Smyth et al., 2020). Following a 20-min wait period, participants provided the final saliva sample (recovery) and then the final state body shame questionnaire. This saliva sample and questionnaire were approximately 50-min post-stressor onset and served as a recovery time point (Dickerson & Kemeny, 2004; Smyth et al., 2020). Finally, a questionnaire asking about previous anthropometric experience was completed. Following the study, participants were asked what they thought the true purpose of the study was and were fully debriefed. At this time, informed consent was obtained. No participants successfully guessed the true purpose of the study or withdrew from the study.

#### High-Threat Condition

The principal researcher (male), a research assistant (female), and two confederates were present in the high-threat condition only. The male confederate (described as a second participant) represented the male ideal (muscular build, well-defined abdominals, strong arms, and a “V” shaped figure; Frederick et al., 2007; Tiggemann, 2011) while the female confederate (described as a second research assistant) represented the female ideal (thin and attractive according to North American standards; Tiggemann, 2011). Testing in the high threat condition took place in front of the two mirrored walls to ensure that the participants were focused on their own physique, as well as those of the confederates, throughout the testing procedure.

Once changed into their shorts and t-shirt, participants were directed to their seat, which was situated immediately to the right of the male confederate. This seating was purposeful as it forced the participant to either look at the two mirrored walls in front of them and to their right, or the male confederate to their left, during the entirety of the testing session. Following the second saliva sample (pre-threat), the female confederate and a research assistant brought out the testing equipment (i.e., skin calipers and measuring tape, handgrip dynamometer, scale, and stadiometer) and set up the video camera. The principal researcher then explained all the testing procedures. Participants were told that they would undergo several physical and strength assessments to indicate overall strength and muscularity, with their shirts off for accuracy. They were told that the video camera was there to ensure the measurements were taken properly and accurately although the camera was not actually recording. Standardized laboratory procedures for all physical and strength measures were used.

The male confederate was always tested first, directly in front of the actual participant. All measurements were taken and read out loud by the female confederate, such that everyone in the room could hear, and were recorded by the principal researcher. Once all the measurements were completed, participants were told that the research assistant was going next door to calculate percent body fat and determine norms for the first participant’s (male confederate’s) values. Upon returning to the room (in approximately 2–3 min) the research assistant passed the “results” to the female confederate, who then read the male confederate’s “results” out loud so all could hear. Values indicated that the male confederate tested in the healthiest range for percent body fat and in the upper range for strength and arm, chest, and waist circumferences, similar to what is typically seen for international level athletes. Following, the participant underwent the same anthropometric measurements in the exact same order. The female confederate read all measurements out loud for everyone in the room to hear. However, upon completion, the participant was told that to save time the testing would proceed, and all measurements would be made available to them at the end of the testing session. Following the fifth saliva sample (recovery) participants completed a questionnaire assessing how similar the male confederate was to their body ideal, and how attractive they found the female confederate.

#### Low-Threat Condition

Testing in the low-threat condition was performed in the same laboratory as the high-threat condition; however, all of the testing was performed away from the mirrored walls. Only the principal researcher and one research assistant were present. Following screening, participants were asked to change into their shorts and t-shirt. The anthropometric tests were the same as those in the high-threat condition but were recorded quietly without any feedback given to the participants and with no video camera present. In this condition, the participant waited quietly for 10 min before completing their anthropometric measurements to account for the time of the confederate’s measurements in the high-threat condition. Due to the removal of the social-evaluative aspects of the design (i.e., mirrors, confederates, additional researchers, video camera, and shirtless anthropometrics with feedback), the low-threat condition acted as the control group.

### Salivary Collection Procedures and Cortisol Assay Determinants

Saliva was collected using Salivettes (Sarstedt). Participants were instructed to accumulate as much saliva in their mouths as possible. They were then told to remove the cap off the plastic tube and place the swab in their mouth without using their hands, hold it in their mouth for 1 min, gently moving saliva toward it without chewing or swallowing it. After 1 min, participants were told to guide the swab from their mouth back into the plastic tube without touching the edges of the container with their lips to avoid any potential cross-contamination of saliva samples. Immediately after testing, the Salivettes were centrifuged at 3,400 rpm at 1,100 × g using the Hamilton Bell V6500 Vanguard and stored in a freezer at −20°C (−4°F) until analysis.

All enzyme immunoassays were carried out on NUNC Maxisorb plates. Cortisol antibodies (R4866) and matching horseradish peroxidase conjugate were obtained from C. Munro of the Clinical Endocrinology Laboratory, University of California, Davis. Steroid standards were taken from Steraloids, Inc. Newport, Rhode Island. Plates were first coated with 50 μL of antibody stock diluted at 1:8500 in a coating buffer (50 mmol/L bicarbonate buffer pH 9.6), then sealed and stored for 12–14 hr at 4°C. A 50 μL wash solution (0.15 mol/L NaCl solution containing 0.5 ml of Tween 20/L) was added to each well to rinse away unbound antibody. Next, a 50 μL phosphate buffer was added to each well. Plates were incubated at room temperature for 2 hr before standards, samples, or controls were added. Two quality control salivary samples, at 30% and 70% binding (the low and high ends of the sensitivity range of the standard curve), were prepared. A 50 μL cortisol horseradish peroxidase conjugate was added to each well, with a 50 μL of standard, sample, or control. Plates remained incubated for 1 hr after plate loading, then washed with 50 μL wash solution and 100 μL of a substrate solution of citrate buffer; H_2_O_2_ and 2,2′-azino-bis (3- ethylbenzthiazoline-6-sulfonic acid) were added to each well. The plates were covered and incubated while shaking at room temperature for 30-60 min. Plates were read with a single filter at 405 nm on the microplate reader (Titertek multiskan MCC/340). Blank absorbances were obtained, standard curves were generated, a regression line was fitted to the sensitive range of the standard curve (typically 40–60% binding), and samples were interpolated into the equation to get a value in pg per well. Each sample was assayed in duplicate, and averages were used.

### Data Analytic Strategy

Prior to analyses, the data were screened to ensure the statistical assumptions were upheld. Following data screening and assumption testing, an independent samples’ *t*-test was used to assess participants’ perceptions of the confederates in the high-threat condition between athletes and non-athletes. In addition, descriptive statistics (means and standard deviations) for the demographic and main study outcomes, split by condition, athlete status and timepoint when appropriate, were generated (Tables 1 & 2). To address the purposes of the current study, two separate analyses were carried out. First, to examine the psychological (i.e., state body-shame) responses to, and recovery from, the body image SET, a two (condition; high vs. low threat) by two (athlete status; athlete vs. non-athlete) by four (time; pre vs. post vs. intermediate vs. recovery) repeated measures ANOVA (RM ANOVA) was performed. Similarly, to examine the physiological (i.e., salivary cortisol) responses to, and recovery from, the body image SET, a two (condition) by two (athlete status) by four (time) repeated measures ANCOVA (RM ANCOVA) was utilized. In this analysis, the covariate included was baseline salivary cortisol.

**Table 1.** Descriptive Statistics by Condition and Athlete Status

Demographic variables	Non-athletes		Athletes	
	Low-threat (*n* = 34) *M (SD)*	High-threat (*n* = 29) *M (SD)*	Low-threat (*n* = 28) *M (SD)*	High-threat (*n* = 21) *M (SD)*
Age	19.76 (2.56)	19.55 (2.06)	19.79 (1.32)	19.76 (1.26)
Height (cm)	176.96 (6.64)	176.69 (7.18)	182.47 (9.18)	182.73 (8.32)
Weight (kg)	74.68 (15.65)	77.4 (13.26)	79.53 (14.74)	84.31 (13.77)
Body fat percentage	14.43 (7.93)	16.62 (8.83)	12.33 (7.1)	12.96 (8.64)
Strength (kg)	77.24 (22.47)	82.55 (18.08)	84.25 (15.81)	104.43 (18.87)
Physical activity	1,388.62 (1,492.04)	1,155.24 (1,463.13)	2,686.43 (1,576.81)	3,001.57 (1,975.93)
MBAS—Muscularity	3.47 (1.06)	3.45 (1.05)	3.51 (1.06)	3.10 (.84)
SATAQ—Muscularity	3.17 (.91)	3.78 (.79)	3.22 (.95)	3.92 (88)

*Note.* Physical activity is reported in MET minutes per week. MBAS—muscularity = concerns about muscularity (range: 1–6); SATAQ—muscularity = internalization of the muscular ideal (range: 1–5).

**Table 2.** Descriptive Statistics for State Body Shame and Salivary Cortisol by Athlete Status and Condition at Each Timepoint

Psychobiological variables	Non-athletes		Athletes	
	Low-threat (*n* = 34) *M (SD)*	High-threat (*n* = 29) *M (SD)*	Low-threat (*n* = 28) *M (SD)*	High-threat (*n* = 21) *M (SD)*
Pre-cortisol	4.61 (3.85)	2.37 (1.76)	3.87 (3)	3.21 (3.11)
Post-cortisol	5.03 (3.85)	2.36 (2.14)	4.41 (3.91)	2.57 (2.16)
Intermediate-cortisol	4.75 (3.67)	3.32 (2.42)	4.02 (3.19)	3.67 (2.6)
Recovery-cortisol	4.16 (3.44)	3.56 (2.06)	4.07 (3.42)	3.33 (1.68)
Pre-WBRSG	.82 (.54)	.64 (.57)	.52 (.59)	.32 (.36)
Post-WBRSG	.74 (.59)	.81 (.75)	.53 (.61)	.53 (.65)
Intermediate-WBRSG	67 (.57)	.68 (.75)	.42 (.5)	.35 (.47)
Recovery-WBRSG	.59 (.59)	.61 (.73)	.42 (.53)	.32 (.41)

*Note.* Cortisol = salivary cortisol; BISS = state body dissatisfaction (range: 1–9); WBRSG = state body shame (range: 0–4).

## Results

Two univariate outliers for physical activity levels were identified, and these values were winsorized (Field, 2013). The assumption of homogeneity of covariance was violated for the repeated measures analysis of variance for body shame. As such, Greenhouse-Geisser values were analyzed (Field, 2013). Assumptions for normality, multicollinearity, linearity between covariates, and independence of covariates and treatment effect were all upheld (Field, 2013).

### Descriptive Statistics and Manipulation Checks

Athletes and non-athletes differed significantly on height (*F*_1,108_ = 5, *p* < .001), weight (*F*_1,108_ = 4.48, *p* = .037), physical activity (*F*_1,108_ = 26.03, *p* < .001), and internalization of the muscular ideal (*F*_1,108_ = 14.81, *p* < .001) with athletes being significantly higher on each measure (Table 1). Athletes in the high-threat condition had significantly greater strength scores compared with the athletes in the low-threat condition and the non-athletes in the low and high-threat condition (*F*_1,108_ = 4.09, *p* = .046). There were no significant differences for the non-athletes in the low- and high-threat condition on height, weight, body fat percentage, strength, physical activity, internalization of the muscular ideal, or male body attitude scores. All other measures were not significantly different.

Athletes (*M* = 3.05, *SD* = .92; *M* = 3.19, *SD* = .60) and non-athletes (*M* = 3.04, *SD* = .85; *M* = 3.14, *SD* = 1.09) in the high-threat group did not differ in their perceptions of the female (*t*[46] = −.041, *p* = .967) and male (*t*[45.24] = −.217, *p* = .829) confederates, respectively. Moreover, both means were well over the half-way point of two. Thus, confederates met/approached Western society’s body ideals and served their role.

Means and standard deviations for the dependent variables at each time point, by condition and athletic status, were calculated (Table 2). Pearson’s bivariate correlations were calculated between height, weight, physical activity, body fat percentage, strength, MBAS—muscularity, SATAQ—muscularity, and baseline cortisol with each of the dependent variables to identify potential covariates. For cortisol, baseline salivary cortisol was identified as a covariate and added to the analysis; however, no covariates were identified for state body shame.

### State Body Shame

A three-way (athlete status, condition, and time) mixed RM ANOVA was produced. There was no significant three-way interaction (*F*2.43,262.57 = .148, *p* = .899, η2 = .001), and no significant interaction between athlete status and condition (*F*1,108 = .115, *p* = .736, η2 = .001). However, there was a main effect of athlete status (*F*1,108 = 6.421, *p* = .013, η2 = .056) with athletes reporting lower levels of body shame overall compared with the non-athletes, regardless of condition. There was also a significant time by condition interaction (*F*2.43,262.57 = 4.58, *p* = .007, η2 = .041). Since there were no significant three- or two-way interactions with athlete status, athletes and non-athletes were combined, and follow-up RM ANOVAs were produced to examine changes over time within in each condition. In the low-threat condition, the RM ANOVA revealed a significant main effect of time (*F*2.56,156.13 = 8.98, *p* < .001, η 2 = .128), and follow-up pairwise comparisons showed significant decreases in state body shame from the pre-threat to intermediate and recovery time points (*p* = .013; *p* = .001, respectively), and from post-threat to intermediate and recovery time points (*p* = .018; *p* = .004, respectively). When examining the high-threat condition, there was a significant main effect of time (*F*2.2,107.54 = 6.56, *p* = .001, η 2 = .118). Follow-up pairwise comparisons revealed significant increases in state body shame from pre- to post-threat (*p* = .05). Follow-ups revealed significant decreases from post-threat to the intermediate and recovery time points (*p* = .022; *p* = .003, respectively).

### Salivary Cortisol

A three-way (athlete status, condition, and time) mixed RM ANCOVA was produced, with baseline salivary cortisol as the covariate. There was no significant three-way interaction (*F*3,321 = .356, *p* = .785, η 2 = .003), and no significant two-way interaction between athlete status and condition (*F*1,107 = .073, *p* = .787, η 2 = .001) or main effect of athlete status (*F*1,107 = .392, *p* = .532, η 2 = .004). However, there was a significant time by condition interaction (*F*3,321 = 3.34, *p* = .02, η 2 = .03). With no significant difference between the athletes and non-athletes, participants were combined, and follow-up RM ANCOVAs were produced to examine changes over time by condition. In the low-threat condition, a significant main effect of time was found (*F*3,180 = 2.766, *p* = .043, η 2 = .044); however, the follow-up pairwise comparisons were not significant. In the high-threat condition, the RM ANCOVA was significant (*F*3,144 = 5.83, *p* = .001, η 2 = .108) and follow-up pairwise comparisons revealed significant increases in salivary cortisol from post-threat to the intermediate timepoint (*p* = .011), and significant increases from post-threat to the recovery timepoint (*p* = .016).

## Discussion

The overall purpose of the current study was to examine psychobiological responses to (i.e., state body shame and salivary cortisol), and recovery from, a social-evaluative body image threat in young men and whether those responses differed by athlete status. It was first hypothesized there would be significant increases in salivary cortisol (pre- to intermediate-timepoint) and state body shame (pre- to post-threat) in the high-threat condition only, with a subsequent return to baseline values 50-min post-stressor onset, with increases greater in the non-athletes compared with the athletes in the high SET condition. Finally, it was hypothesized that there would be no significant changes in non-athletes’ and athletes’ psychobiological responses in the low SET condition. These hypotheses were partially supported; participants in the high-threat condition exhibited significant increases in state body shame and salivary cortisol immediately following the threat, with a subsequent return to baseline values for state body shame; however, contrary to the hypotheses, there were no significant differences between the non-athletes’ and athletes’ responses.

### Athletes Versus Non-Athletes

Contrary to both body image (Hausenblas & Symons Downs, 2001; Varnes et al., 2013) and SSPT (Rimmele et al., 2007, 2009) research, athletes and non-athletes did not differ significantly in terms of their state body shame and salivary cortisol responses to the body-specific SET. Studies have reported that athletes tend to exhibit low levels of body dissatisfaction compared with non-athletes (Hausenblas & Symons Downs, 2001), and SSPT research has supported the idea that athletes exhibit dampened psychological responses to psychosocial stressors compared with non-exercisers. Specifically, Rimmele et al. (2007, 2009) reported that untrained men had significantly worsened mood and calmness, and greater anxiety, compared with elite athletes following a psychosocial stressor (i.e., TSST). Although the current study was the first study to examine state body shame responses in athletes, it was expected that state body shame would follow a similar pattern as other psychological responses (i.e., calmness, mood, and anxiety) with athletes exhibiting smaller changes in shame following a social-evaluative body image threat compared with non-athletes. However, there are some potential reasons as to why athletes in this study exhibited similar psychobiological responses compared with the non-athletes.

This is the first study to examine body shame responses in athletes versus non-athletes (Rimmele et al., 2007, 2009; Rohleder et al., 2007). It is possible that athletes are trained to manage commonly occurring emotions, such as anger, anxiety, and calmness, in high-stress situations compared with more complex emotions (i.e., self-conscious emotions), such as shame (Martinent et al., 2015). In addition, research has reported that emotions serve specific goals. Basic emotions serve survival and reproductive goals while self-conscious emotions serve social goals, such as avoiding social exclusion and protecting from social evaluation (Tracy & Robins, 2004). To feel shame, individuals have to internalize a societal standard and reflect on the discrepancy present between the self and the societal ideals (Tracy & Robins, 2004). Shame is elicited in response to this discrepancy and social evaluation to bring attention to the discrepancy such that action can be taken to mitigate it. Thus, while other studies demonstrated significant differences between athletes and non-exercisers, they examined basic emotions (e.g., anxiety, calmness, mood) rather than self-conscious emotions, which specifically serve to protect against social evaluation and threats to the social self (Dickerson et al., 2004; Dickerson & Kemeny, 2004; Kemeny et al., 2004; Rimmele et al., 2007, 2009; Tracy & Robins, 2004). Shame and body-related shame have never previously been examined in the context of athletic status and it is possible that it would have significantly increased in response to SETs across all previous studies regardless of athlete status or competition level. Moreover, body image SETs may be more salient for athletes compared with other types of SETs (i.e., TSST) given the greater body-related pressures and internalization of the muscular ideal they experience (Petrie & Greenleaf, 2012). For these athletes, commonly used psychosocial stressors may not challenge the social self to the same degree as body image SETs.

Second, the varsity athletes in the current study were at a lower competitive level (e.g., interuniversity) compared with previous studies that examined more elite level athletes (e.g., national endurance athletes), thus potentially reporting greater psychological responses. Rimmele et al. (2007, 2009) examined nationally competing endurance athletes and compared them with men who reported training <2 hr per week. Rimmele et al. (2009) reported significantly higher levels of state anxiety between the elite athletes and non-athletes but no significant differences in state anxiety or salivary cortisol between the amateur athletes and non-exercisers. It could be argued that most university varsity athletes are more accurately categorized as amateur level athletes as opposed to elite level athletes (Swann et al., 2015), with few exceptions, due to the frequency with which they compete at a national and international level, years of experience within the sport, and training frequency. Thus, it is possible that athletes in the current study reported state body shame and salivary cortisol responses more similar to amateur level athletes and non-exercisers in previous studies (Rimmele et al., 2009).

Finally, the non-athletes in the current study reported high levels of physical activity and met recommended weekly physical activity guidelines for adults (WHO, 2022). As such, the non-athletes in this study may have exhibited blunted psychological responses, similar to those of the amateur varsity athletes. Therefore, it is possible that differences between athletes and non-athletes were not found due to a very active control group and an athletic population competing at a lower level of competition compared with previous studies (Rimmele et al., 2007, 2009).

These studies together help to highlight the complicated relationship that exists between athletes and their body image and supports the existing evidence suggesting that athletes experience body image concerns and respond to social evaluation of the physique (Galli & Reel, 2009; Lunde & Gattario, 2017).

### Implications

The current study provides a number of important implications for future body image and SSPT research. First, it offers further support for the idea that male athletes and non-athletes experience meaningful and impactful body image concerns comparable to women (Chapman & Woodman, 2016; Fallon et al., 2014; Galli & Reel, 2009), and that further research is needed to better understand the nature and magnitude of these concerns, as men have disproportionately been neglected within the body image research. Second, body image research that has previously compared athletes on sport-related characteristics (e.g., ball-sport vs. endurance sport) has primarily been conducted with U.S. athletes and has frequently been inappropriately generalized to other contexts (e.g., university athletes in other countries such as Canada; Hausenblas & Symons Downs, 2001). Cultural differences surrounding university sports, as well as differences in available resources due to large financial investments in athletics, create vastly different lived experiences (e.g., pressures to perform, scholarship opportunities, work–school–life balance) for American NCAA athletes compared with Canadian varsity athletes. Thus, generalizing many psychosocial findings, such as those pertaining to body image, from National Collegiate Athletic Association (NCAA) to Canadian varsity level athletes is likely inappropriate. This study helps to highlight the complex relationship that exists between male athletes and their body image and provides evidence for the careful consideration of sport type, competition level, and country when examining body image concerns in university athletes.

Within the context of SSPT, this study provides further evidence for the integration of SSPT within a body image context by demonstrating the impact of body image SETs on non-athletes’ and athletes’ psychobiological responses. Much of the SSPT literature has utilized performance-based SETs (i.e., speech tasks; Dickerson & Kemeny, 2004); however research over the last decade, as well as the current study, adds to the growing body of research that utilizes body-specific SETs to study the acute effects and consequences of social-evaluation of the physique (Cloudt et al., 2014; Lamarche et al., 2016, 2017; Martin Ginis et al., 2012; Smyth et al., 2020). Future studies should continue to examine a wider variety of SETs that are relevant to the populations of interest. Finally, these findings provide further support for the notion that *actual* (not anticipatory) body image stressors may be required to examine physiological changes (Lamarche et al., 2016, 2017; Smyth et al., 2020).

The current study provides evidence of the physiological consequences of acute body image stressors involving social evaluation in male athletes. These social-evaluative body image threats, and subsequent physiological responses, may frequently occur within Western society as Western culture tends to place great importance and pressure on people and athletes to meet body image ideals (Galli & Reel, 2009; Lunde & Gattario, 2017; Petrie & Greenleaf, 2012; Tiggemann, 2011). Failure to meet these ideals can result in social exclusion (Westermann et al., 2015) and ridicule (Markham et al., 2005), which may lead to increased feelings of body-related shame and subsequent cortisol responses. These body image stressors exist within sport, though they differ slightly depending on sport type and athlete status (Galli & Reel, 2009; Lunde & Gattario, 2017; Varnes et al., 2013), and findings from the present study suggest that athletes and coaches should be aware of the existence and potential consequences of body-related comments, stressors, and evaluation within the university sporting context. These body image stressors, when accompanied by social evaluation, can evoke feelings of shame—including body shame—which may lead to avoidance behaviors (Leary, 1992) and negatively influence the experiences of athletes in their given sport. In addition, negative body-related stressors may accumulate over time in both athletes and non-athletes leading to lasting negative effects on self-esteem (Fox, 1999).

### Limitations and Future Directions

*This study* is the first to examine psychobiological responses to (i.e., body shame and salivary cortisol), and recovery from, a SET in university athletes from non-aesthetic sports and non-athletes. Moreover, this is the first study to examine these types of responses to a body-specific SET and fills a gap from previous studies (Lamarche et al., 2016, 2017; Smyth et al., 2020) that excluded athletes due to their unique body image concerns. Despite these strengths, the study is not without limitations. Importantly, the sample of participants was comprised of predominantly heterosexual White men, and findings cannot be generalized to those identifying as other sexual orientations or ethnicities. Another limitation is that including both varsity and club sport athletes from Canadian universities may have confounded competition level. Certain university sports compete provincially, while others have the opportunity to compete nationally. In addition, certain sports, such as ball hockey, ultimate frisbee, and dragonboat do not currently compete at either level. Thus, differences in competition level may influence body image pressures and concerns, and consequently psychobiological responses to body image SETs. The current study recruited men from non-appearance focused sports to control for the potential impact of sport type on psychobiological responses to the body image SET. Researchers have reported that athletes in weight-dependent and appearance-focused sports tend to exhibit higher negative body image and disordered eating tendencies compared with non-appearance-focused sport athletes (Chapman & Woodman, 2016; Galli & Reel, 2009; Varnes et al., 2013). Thus, it is possible that individuals participating in weight-dependent or appearance-focused sports would exhibit heightened psychobiological responses to the body image SET and future research should examine the potential impact of sport type.

Another limitation is with our physiological measure of stress. Cortisol is a relatively unstable hormone that can be affected by many factors (e.g., diurnal rhythms, individual differences such as nutrition, physical activity, trait anxiety, and depression, perception of stressful stimuli). Although measures were taken to reduce outside factors that can affect cortisol (i.e., ensuring no stressful events occurred on the day of testing; testing between 2 and 7 pm when cortisol is at its lowest and most stable levels; Dickerson & Kemeny, 2004), it is possible that individual factors, like anxiety and depression, may have affected cortisol (Burke et al., 2005). Next, while the athletes reported significantly greater levels of physical activity, the non-athletes in the study were also very active, on average meeting physical activity MET minute recommendations per week (WHO, 2022). It is possible that the athletes and non-athletes were too similar in terms of physical activity for any significant differences to be found (Mücke et al., 2018; Wunsch et al., 2019).

Moreover, the recommended sample size as determined by a priori power analysis was based on medium to large effect sizes found in previous studies that investigated only two-way interactions (i.e., time by condition). The current study was most likely underpowered to investigate potential three-way interactions (i.e., time by condition by athlete status), as indicated by the low observed powers. However, observed power for the two-way interactions (i.e., time by condition) was sufficient.

Researchers should continue to examine psychobiological responses to body image SETs in more diverse samples to extend findings to other populations. Gay men typically report higher body image concerns compared with heterosexual men (Morrison & McCutcheon, 2011). In addition, Black individuals generally report higher positive body image and lower negative body image compared with White samples (Bruns & Carter, 2015; van den Berg et al., 2010). Thus, it is possible that gay men may report higher psychobiological responses compared with their heterosexual counterparts, and that non-White participants could exhibit blunted responses. Researchers should compare psychobiological responses to body image SETs in athletes of different competitive levels to examine the unique influence of competition level within a Canadian university context. No studies, thus far, have examined the effect of competition level on the psychobiological responses to body image SETs of athletes.

## Conclusion

The current study examined psychobiological responses to social-evaluative body image threats in male university athletes and non-athletes. Male athletes and non-athletes exhibited similar increases in state body shame and salivary cortisol in response to the high social-evaluative threat, with a return to baseline levels within 1 hr of the stressor onset. These results are partially consistent with previous literature suggesting that shame and cortisol are unique responses to social evaluation and that amateur athletes exhibit similar responses compared with non-athletes (Dickerson et al., 2004; Dickerson & Kemeny, 2004; Rimmele et al., 2007, 2009). These findings are consistent with previous body image literature, such that body-specific SETs increase body shame (Lamarche et al., 2017) and salivary cortisol (Lamarche et al., 2016, 2017; Smyth et al., 2020). Overall, these findings suggest that male university athletes are not immune to body image concerns or social evaluation of the physique and thus should continue to be investigated in a body image context. Future studies should continue to examine psychobiological responses in male and female athletes, between sport types (e.g., appearance focused vs. non-appearance focused vs. weight category), and over different levels of competition (i.e., recreational and competitive intramural sports vs. OUA vs. Usports), to further examine their effects on these responses.
